# QM-sym, a symmetrized quantum chemistry database of 135 kilo molecules

**DOI:** 10.1038/s41597-019-0237-9

**Published:** 2019-10-18

**Authors:** Jiechun Liang, Yanheng Xu, Rulin Liu, Xi Zhu

**Affiliations:** 1 Shenzhen Institute of Artificial Intelligence and Robotics for Society (AIRS), 2001 Longxiang Road, Longgang District, Shenzhen, Guangdong 518172 China; 2 School of Science and Engineering, The Chinese University of Hong Kong, Shenzhen, 2001 Longxiang Road, Longgang District, Shenzhen, Guangdong 518172 China

**Keywords:** Chemical engineering, Quantum chemistry

## Abstract

Applying deep learning methods in materials science research is an important way of solving the time-consuming problems of typical ab initio quantum chemistry methodology, but due to the size of large molecules, large and uncharted fields still exist. Implementing symmetry information can significantly reduce the calculation complexity of structures, as they can be simplified to the minimum symmetric units. Because there are few quantum chemistry databases that include symmetry information, we constructed a new one, named QM-sym, by designing an algorithm to generate 135k organic molecules with the *C*_*n*_*h* symmetry composite. Those generated molecules were optimized to a stable state using Gaussian 09. The geometric, electronic, energetic, and thermodynamic properties of the molecules were calculated, including their orbital degeneracy states and orbital symmetry around the HOMO-LUMO. The basic symmetric units were also included. This database p rovides consistent and comprehensive quantum chemical properties for structures with *C*_*n*_*h* symmetries. QM-sym can be used as a benchmark for machine learning models in quantum chemistry or as a dataset for training new symmetry-based models.

## Abstract (summary)

**Table:** 

Measurement(s)	Chemical Properties • molecular structure data
Technology Type(s)	quantum chemistry computational method • ab initio quantum chemistry computational method
Factor Type(s)	atom

Machine-accessible metadata file describing the reported data: 10.6084/m9.figshare.9929633

## Background & Summary

Designing novel molecules and structures with specific physicochemical properties is attractive for researchers, and many methodologies are focused on this field. Among these methodologies, high-throughput screening has been proposed as the most straightforward approach^1^, but this method came with a presumption that all the approximations and assumptions made for the adopted modelling techniques are applicable for all stable structures in the entire chemical space^2^. Many quantum chemistry databases have been constructed and reported, including QM7^3,4^, QM7b^5^, and QM9^6^. Among these databases, the QM9 database, which includes the first 134k molecules of the chemical universe GDB-17 database, is the most widely used one in chemistry deep learning applications. All molecules in QM9 are reported along with 15 properties obtained at the B3LYP/6-31G(2df,p) level of theory to reach a higher accuracy compared to experimental values^7^, including the primary energies, enthalpy, and bandgap. The inclusion of these properties makes the database suitable for the small and basic de novo design of new molecules, but the molecules in QM9 consist of at most 9 heavy atoms, which makes the molecule size too small for predicting large molecules, including proteins and polymers. Moreover, there is no symmetry information inside the database. Some essential properties, such as the point group, orbital degeneracy, and selection rules for excitation, are also missing, which makes it impossible to derive excitation events from the QM9 database. In addition, many unstable computer-generated structures containing long N-N chains are included. Due to their low stability and high endothermic properties, long N-N chains tend to decompose and eliminate *N*_2_ ^8^. To identify all of these molecules, we selected all the molecules in the QM9 database with nitrogen chains of more than two nitrogen atoms. All these suspiciously unstable molecules are available in the Supplementary Information, along with their xyz files.

In this work, we provide a dataset of larger symmetrical structures^9^. The benefits of the implemented symmetric properties include considerably reducing the ab initio complexity, and the new database offers the possibility of symmetry recognition by the deep learning architecture to construct a connection between a basic symmetric unit and a conventional structure. Our symmetrical database (QM-sym) includes 135 k organic structures with H, B, C, N, O, F, Cl, and Br atoms. All of them have symmetries other than C1, including C2h, C3h, and C4h. Information about the basic symmetric unit and symmetry centre is also recorded in this database. The structure of this work is organized as follows. We first introduce the methods by which the database is generated and the major difference from the previous QM9 database and discuss some general results. Then, we randomly sample 100 molecules from the QM-sym database and discuss the benchmark with other numerical methods, such as G4MP2^7^, G4^10^, and CBS-QB3^11^, with the same validation as the QM9 database. The QM-sym database, which can serve a function similar to that of the previous ones, provides an efficient training and evaluation database for the data-driven-based machine learning (ML) models in quantum chemistry^12^. Because of the enclosed symmetrical information, the QM-sym database can provide more applications than precious databases in the orbital symmetry-dependent properties, such as excitation degeneracy and the selection rules of transitions. This symmetry-enclosed database can benefit more from the understanding and discovery of structure properties from the ML point of view^13^.

## Methods

### Generation of atomic coordinates

**Fig. 1:**
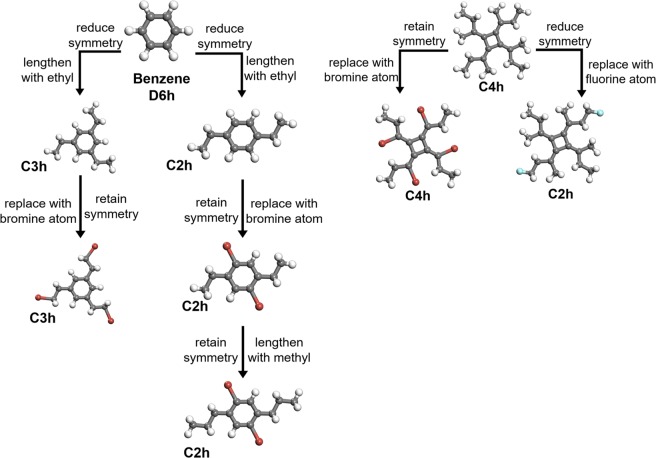
Generation map of some molecules in the QM-sym database by reducing or retaining symmetry through replacements. The left part is the generation map of some C2h and C3h molecules. Starting from benzene (D6h), the symmetry group can be reduced to C2h or C3h or be retained correspondingly depending on whether replacing or lengthening is carried out during generation. The right part is a generation map of some C2h and C4h molecules. Starting from a C4h molecule, the symmetry group can be reduced to C2h by replacing two atoms or be retained by replacing four atoms. Grey, white, red, and blue balls denote carbon, hydrogen, boron, and fluorine atoms, respectively.

The generation of the QM-sym database includes two steps. First, we construct the raw molecular structures based on typical molecular information, such as bond angle and bond length, and then grow them with a genetic algorithm to find a relatively stable structure with given symmetric point groups. Examples of the generation map are shown in Fig. 1. For simplification, we initiate 3 point groups, C2h, C4h, and D6h, which correspond to the ethane, cyclobutene, and benzene, respectively, and then extend the molecular information by adding aliphatic hydrocarbon chains to branches. As shown in the left part of Fig. 1, CH_3_CH_2_ radicals extend the original benzene molecule from the hydrogen sites in two different ways, maintaining the C3h and C2h point groups throughout this step.

Furthermore, to increase the number of structures and structure diversity, we can also randomly sample the halogen elements (F, Cl, and Br) to replace the hydrogen atoms in the corresponding carbon chains and ring motifs. In each sampling, the point group can be retained or reduced by a corresponding partial replacement, such as from C4h to C2h, as shown in the right part of Fig. 1. Additionally, the implemented symmetry can simplify the input of the molecular structure by the primary structure information. For example, benzene, C_6_H_6_, can be represented as CH under the D6h point group.

The QM-sym database records the primary structure information for future symmetrical applications as well. After the raw molecular structures are established, similar to the QM9 database, each of the structures was further precisely optimized at the B3LYP/6-31G(2df,p) level of theory by Gaussian 09^14^ and was set to strictly follow its designed symmetric group, but some of the raw structures generated by the first step were rational in only geometry without any guarantee in chemistry, which would cause SCF convergence to take a long time or even fail. In addition, more structures would be locally trapped in saddle points during the structure optimization due to the complexity and the initial symmetry settings. To solve this problem, we applied a similar strategy to that applied in the QM9 dataset^6^. We chose 200 maximal SCF cycles for all the molecule structures, and for those structures that failed the SCF convergence after 200 steps in 1 cycle, we applied very tight convergence criteria for the Gaussian 09 input and restarted. If the SCF convergence still failed, we ignored those structures. The frequency calculations were included, and for the molecules with imaginary frequencies after the procedure discussed above, we further applied additional iterations by using keywords, opt (calcfc, maxstep = 5, maxcycles = 1000). We retained only the molecular structures with good SCF convergence in the ground state and non-negative frequencies.

The molecular structure with the D6h point group derived from benzene is excellent in both SCF convergence and frequency distribution. It is observed that the DFT calculation can be well accelerated by the symmetrized molecular structures. For the molecular orbitals, we calculate and record at least 5 orbitals upward and downward from the LUMO and HOMO, i.e., from HOMO − 5 to LUMO + 5. The number of contained orbitals depends on the degeneracy; for example, if there is degeneracy between HOMO-6 and HOMO − 5, we count in HOMO-6 as well. A similar operation is performed from the LUMO to the LUMO + 5.

## Data Records

**Fig. 2:**
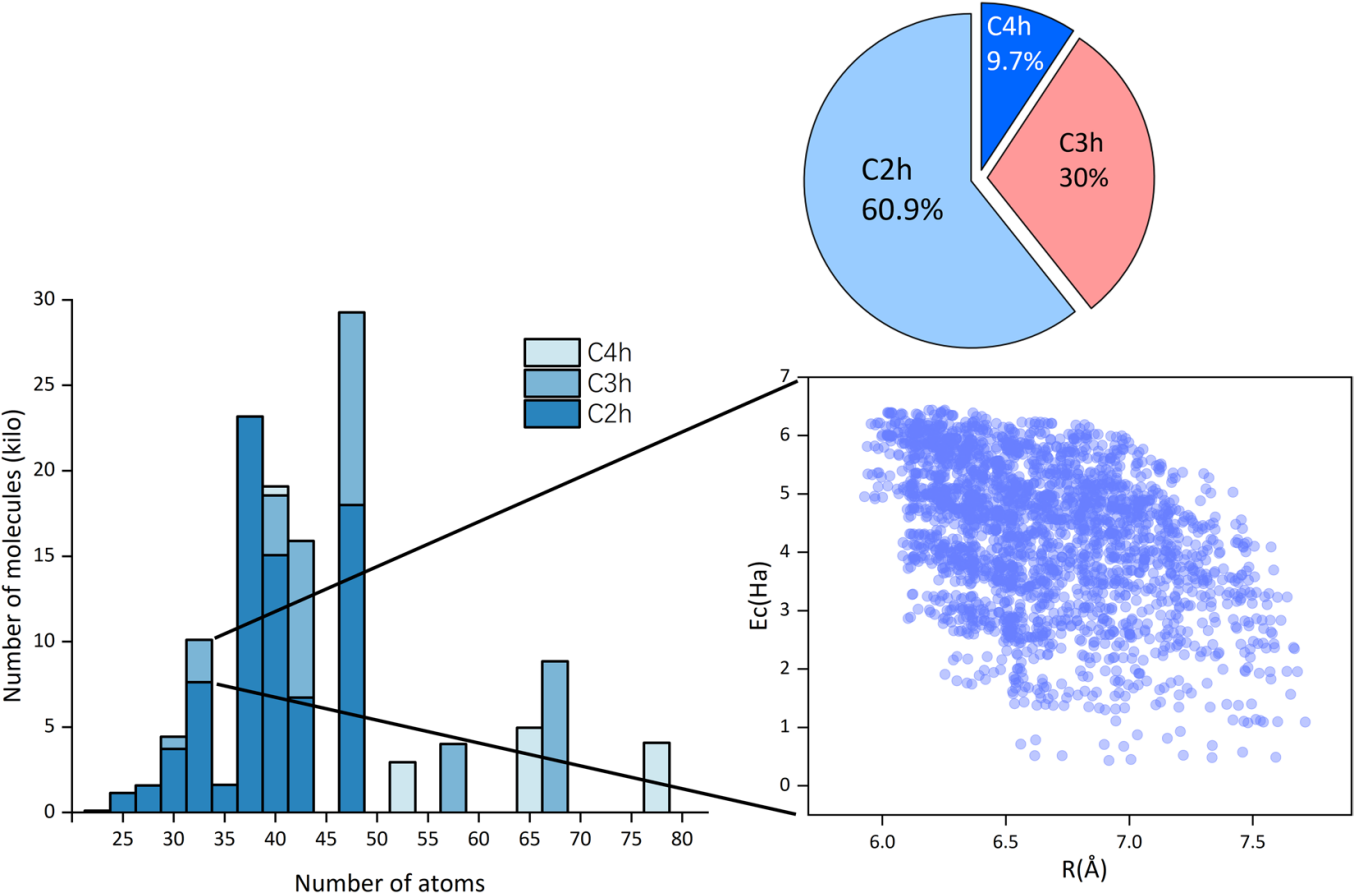
An overview of the QM-sym database. The proportion of each space group in the QM-sym database is shown on the top. The left inset indicates the distribution of molecules with respect to their size according to the number of atoms and space groups. The right inset corresponds to some of the *C*_3_*h* molecules, with their radius of rotation plotted versus their cohesive energy, and shows a distinct tendency of the cohesive energy to decrease when the rotation radius increases.

The QM-sym database is publicly available at GitHub and Figshare^9^ (see the Code Availability part below). It now includes 13.5 k molecular structures (QM_sym_xyz_number.tar) and all the properties, including the point group, information on the basic symmetric unit, enthalpies, atomization, zero-point energy, energy and symmetry labels from at least HOMO − 5 to LUMO + 5. Detailed information on the available properties is documented in the README file. The proportion of each space group is shown in Fig. 2. Additionally, the 100 randomly chosen structures, benchmarked with other numerical methods (G4MP2^7^, G4^10^, and CBS-QB3^11^), are also included in the database as benchmarked.tar.gz. The data are shown in Table 1, and the QM9 benchmark is also included with the results derived from the G4MP2, G4, and CBS-QB3 methods. We randomly choose 100 molecules from 135k molecules and calculate the errors relative to the individual method, identified by the mean absolute error (MAE), root-mean-square error (RMSE), and maximal absolute error (maxAE). The units are kcal/mol. The values from the QM9 database are in parentheses and derived directly from the literature^6^.

**Table 1:** The benchmark of atomization enthalpies at B3LYP/6-31G(2df, p) level compared.

Reference	MAE	RMSE	maxAE
G4MP2	6.1 (5.0)	7.3 (6.1)	18.2 (16.0)
G4	5.4 (4.9)	6.3 (5.9)	15.4 (14.4)
CBS-QB3	5.6 (4.5)	6.7 (5.5)	16.7 (13.4)

The data in the parenthesis are from QM9 database.

### File format

The QM_sym.xyz file contains the atomic coordinates, together with predicted properties information from the Gaussian09 calculation; each structure is indexed by QM_sym_i.xyz, where i is the index of the structure ordered in the database. The xyz file format is one of the most common file formats for molecular chemistry; it is ASCII coded and can be viewed by many free software like VESTA^15^ and Jmol^16^. The basic outline of the xyz format is shown in Table 2.

**Table 2:** xyz file format for molecular structure and properties. The coordinate lines are shown in this format: atom, x position, y position, z position, charge.

Line	Content
1	Number of atoms *N*⋅*n*_*a*_ (+1)
2	Properties of molecule
3, …, 2 + *n*_*a*_	Coordinates of atoms in the first subgroup
3 + *n*_*a*_, …, 2 + 2⋅*n*_*a*_	Coordinates of atoms in the second subgroup
…	…
3 + (*N* − 1)*n*_*a*_, …, 2 + *N*⋅*n*_*a*_	Coordinates of atoms in the last subgroup
(3 + *N*⋅*n*_*a*_ *to end*)	(Coordinates of atom on rotation axes)

The original xyz file format includes only the structure information. Here, in the QM-sym database, we add more property and symmetry information to the comment lines. The user can directly read out all the information from the modified xyz file, as indicated in Tables 2 and 3. *N* in the tables is the number of subgroups, depending on the symmetry *C*_*N*_, and *n*_*a*_ is the number of atoms in each subgroup. ‘+1’ and ‘4 + *N*⋅*n*_*a*_’ in brackets will be present in the xyz files when there are atoms on rotation axes. An indication of the coordinates is shown at the end of the property line. Take ‘11 12 13 14 15 16 17 18 19 110 21 22 23 24 25 26 27 28 210 29 011’, which is a list of ‘signs’, from a C2h structure as an example. The order of signs is the same as the order of the subsequent atom coordinates. Each sign is composed of two parts: a subgroup ID and a position ID. Taking ‘110’ and ‘210’ as an example, the first numbers, ‘1’ and ‘2’, denote that these atoms belong to subgroups 1 and 2, respectively, which have the same primitive structure as the two ‘CH’ subgroups in benzene (C_6_H_6_). The ‘10’ is the position ID for these atoms, which means that ‘110’ and ‘210’ are atoms of the same element at the same position in different subgroups. To differ atoms on rotation axes that belong to all subgroups, we use ‘0’ to denote the subgroup ID and a number that does not belong to any other atom to show its position. The atom coordinates are below the comment line, and the coordinates of the atoms are followed by the Mulliken charges.

**Table 3:** Calculated properties. Properties are stored in the order given by the first column. The orbital degeneracy and symmetry are from ‘HOMO − 5’ to ‘LUMO + 5’, see further explanation in the following.

No.	Property	Unit	Description
1	*S*	/	Symmetry group
2	*E*_*g*_	eV	Bandgap
3	*E*_*c*_	eV	LUMO
4	*E*_*v*_	eV	HOMO
5, 6, 7	*B*_*v*_	GHz	Rotational constant
8, 9, 10, 11	μ	D	Dipole moment
12	α	*a*_0_^3^	Isotropic polarizability
13	*R* ^2^	au	Electronic spatial extent
14,15	*ε*_v_	J/mol; Kcal/mol	Zero-point vibrational energy
16	*ε*_0_ + *ε*_*ZPE*_	Ha	Sum of electronic and zero-point energies
17	*ε*_0_ + *E*_*tot*_	Ha	Sum of electronic and thermal energies
18	*ε*_0_ + *H*_*corr*_	Ha	Sum of electronic and thermal enthalpies
19	*ε*_0_ + *G*_*corr*_	Ha	Sum of electronic and thermal free energies
20	*C*_v_	*Cal* · *Mol-Kelvin*^−1^	Heat capacity
21–32	*D*	/	Degeneracy of orbitals
33–44	*S*_*o*_	/	Symmetry of orbitals
45-(*N*⋅*n*_*a*_ + 44)	*S*_*g*_	/	Indication of subgroups and atoms

### Properties

The properties are numerically derived from the DFT calculation after the full relaxation of the molecular geometry with the initial symmetry settings. The technical details of the DFT calculation can be expressed as B3LYP/6-31G(2df,p), with a 10^−5^ eV criterion for energy convergence. The initially given symmetry information and all the other properties of the 135 k molecular structures are listed in Table 3. Compared with the QM9 database, the QM-sym database is supplemented with the orbital symmetry and eigenvalue information from ‘HOMO − 5’ to ‘LUMO + 5’ and the symmetric unit cell segments by the point group. According to the symmetry information above, the user can calculate the excitation transition probability from ‘HOMO − 5’, …, HOMO to LUMO, …, ‘LUMO + 5’. To simplify the expressions of orbitals, in the xyz file, we regard the degenerated orbitals as one sign. For example, ‘1|2|1|1|BU|BG|AU|AG’ contains 5 orbitals, and there are two orbitals with ‘BG’ in the symmetry that are degenerated. Since all the molecules and the properties are symmetrized, a symmetrized neural network can be used to further optimize the efficiency from the symmetrical input^17^.

## Technical Validation

### Validation of geometry and symmetry consistency

**Fig. 3:**
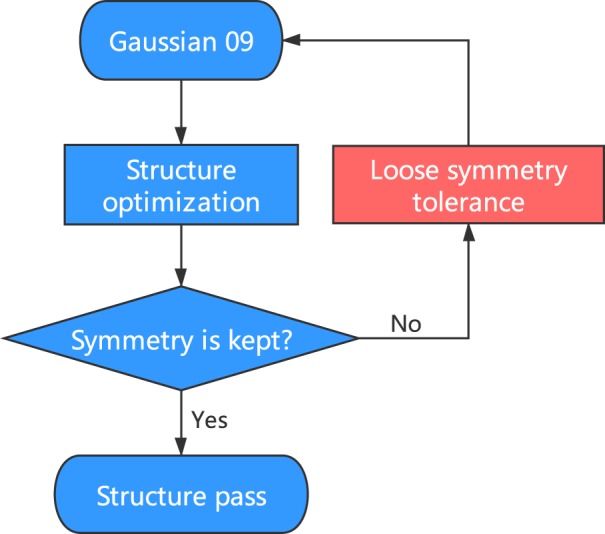
Flow chart of the geometry check.

In the QM9 database^6^, the authors applied InChI (IUPAC International Chemical Identifier) strings^18^ and SMILES (simplified molecular-input line-entry system) strings^19^ for the forward-feedback double-check with semi-empirical methods (SEMs) such as PM7 in MOPAC^20^. Since both the InChI and SMILES descriptions lose geometric information regarding the bond lengths, bond angles, and dihedral angles, there are approximately 3000 structures that fail the consistency check in QM9. Here, in the QM-sym database, the arrangements of the elements are all constrained by the given point group; i.e., the symmetry can be the only measurement of the geometry. Due to the initial setting of the symmetry, both the DFT and SEM calculations are initially fully symmetrized in the given point group. For some structures, the symmetry information may be lost during the structure optimization, causing geometric inconsistency. The flow chart of the symmetry check is shown in Fig. 3. This issue can be solved by setting a ‘loose’ criterion for the symmetry identification and redoing the Gaussian 09 calculation. We find that there are approximately 2000 structures that fail the symmetry invariant test out of the 135 k molecules in the database. When we look closely into the 2000 structures by distributing the atomization energy and element distribution (except for hydrogen and carbon), we find that most of the structures that fail the symmetry check are of low stability with unphysical chemical structures; thus, we do not add them to QM-sym.

### Validation of the quantum chemistry results

In the QM-sym database, as discussed above, all 135 k molecules are first generated by the symmetry operation; then, the structures are optimized at the B3LYP/6-31G(2df, p) level of DFT, with the same theory quality as that in the previous QM7^3,4^, QM7b^5^, and QM9^6^ databases. The additional benchmarks with the G4MP2, G423, and CBS-QB3 functions are summarized in Table 1 as well. The QM-sym database arrives with an accuracy comparable to that of QM9, as the atomization enthalpies in the benchmark belong to the scalar properties and there is no significant benefit gained from accuracy in this domain. For all three additional functions, within the 100 randomly selected molecules, the MSE is approximately 6.1 kJ/mol, and the RMSE is 6 kJ/mol. In addition to the low numerical errors, the orbital degeneracy and symmetry-dependent calculation, similar to the transition selection rules, obtains the exact results.

### Spectral transition probability

**Fig. 4:**
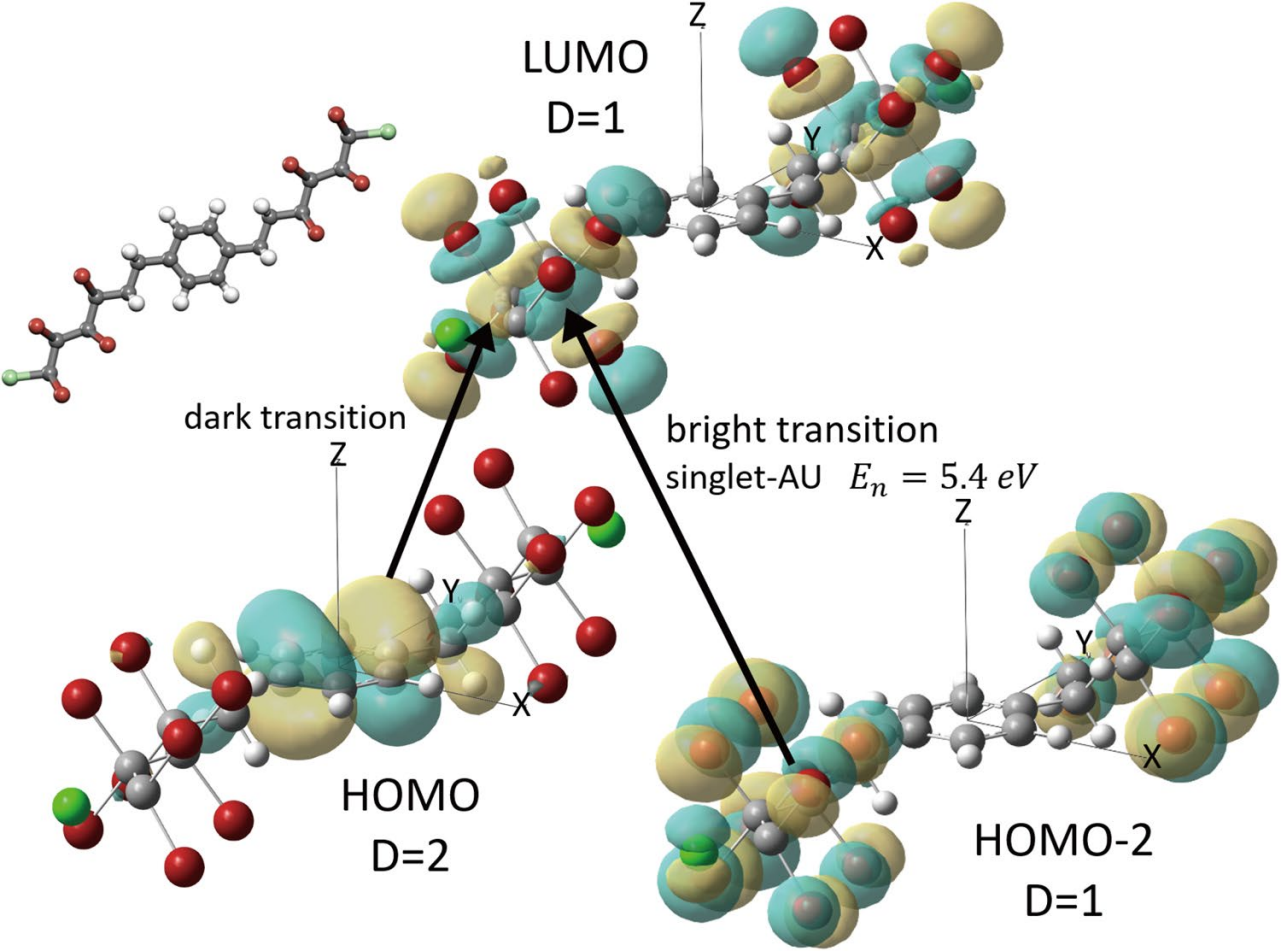
Sketch of the excitations between orbitals with different energy levels. The degeneracy levels of the *HOMO*, *HOMO* − 2 and *LUMO* are 2, 1 and 1, respectively. From the results of both group theory and Gaussian 09, the transition from the *HOMO* to the *LUMO* is dark, while that from *HOMO* − 2 to the *LUMO* is bright, with a singlet with AU in terms of symmetry and an energy of 5.4 eV. An example molecule used for the spectral transition probability calculation is shown at the top left.

With the database present, according to methods provided by F. Albert Cotton^21^, the spectral transition probability could also be calculated based on the symmetry of the orbitals. By defining symmetry operations of the initial and targeting orbitals *ψ*_*i*_, *ψ*_*j*_, and the transition moment operator *μ*, the intensity of the transition is given by the equation:

1 $$I\propto \int {\psi }_{i}\otimes \mu \otimes {\psi }_{j}d\tau $$

where ‘⊗’ refers to the direct product of symmetry operations. The characters of the representation of a direct product are equal to the products of the characters of the representations based on the individual sets of functions. Only when the total symmetry operation is present in the result of the direct product *ψ*_*i*_ ⊗ *μ* ⊗ *ψ*_*j*_ will this integral be nonzero; i.e., the transition of electrons from the *ψ*_*i*_ orbital to the *ψ*_*j*_ orbital via operator *μ* is possible. According to the results of Gaussian 09, part of the energy level diagram of the C2h molecule in Fig. 4 is also shown in Fig. 4 as an example. To check the spectral transition, the operator *μ* must contain the Cartesian coordinates *x*, *y* or *z* (full character table for C2h is available in Table 4). In this case, for *x* or *y* polarized light, *μ* = *Bu*; for the *z* polarized light, *μ* = *Au*. Details regarding the characters calculated via Eq. 1 are shown in Table 5.

**Table 4:** Character table of the symmetry group *C*_2h_.

*C*_*2h*_	*E*	*C*_*2*_	*i*	*σ*_*h*_	*linear*
*A*_g_	*1*	*1*	*1*	*1*	*R*_*z*_
*B*_g_	*1*	*−1*	*1*	*−1*	*R*_*x*_ *, R*_*y*_
*A*_u_	*1*	*1*	*−1*	*−1*	*z*
*B*_u_	*1*	*−1*	*−1*	*1*	*x*, *y*

It could be observed that *x* and *y* polarized light would be yielded by the symmetry operation *B*_*u*_ and that *z* polarized light would be yielded by *A*_*u*_.

**Table 5:** Probabilities for the transition from *T*_*i*_ to *T*_*f*_, calculated using direct products.

*T*_*i*→ *f*_	*Polarization of light*	*Direct products*	*Resulting characters*				*P*_*T*_
			*E*	*C*_2_	*i*	*σ*_*h*_	
*T*_*HOMO*→ *LUMO*_	*x*, *y*	*B*_*g*_ × *B*_*u*_ × *A*_*g*_	1	−1	−1	1	×
	*z*	*B*_*g*_ × *A*_*u*_ × *A*_*g*_	1	1	−1	−1	×
*T*_*HOMO*−2→ *LUMO*_	*x*, *y*	*A*_*u*_ × *B*_*u*_ × *A*_*g*_	1	−1	1	−1	×
	*z*	*A*_*u*_ × *A*_*u*_ × *A*_*g*_	1	1	1	1	√

Only the last one is bright since only the direct product *A*_*u*_ × *A*_*u*_ × *A*_*g*_ contains the total symmetry *A*_*g*_. *P*_*T*_ is the probability of each transition, the transition is bright with √ and dark with ×.

From the decomposition formula *a*_*i*_ = Σ_*R*_*χ*_*r*_(*R*)*χ*_*i*_(*R*)/*h* (*R* refers to all the symmetry operators of the symmetry group, *χ*_*r*_ refers to the character of the reducible representation Γ_*r*_, *χ*_*i*_ refers to the characters of the *i*th irreducible representation, *h* refers to the dimensions of the symmetry group, and *a*_*i*_ refers to the number of times the *i*th irreducible representation occurs in Γ_*r*_)^21^, it could be concluded that the total symmetry operation *A*_*g*_ occurs only in the transition *T*_*HOMO*−2→*LUMO*_; thus, this is the spectral transition of this molecule with the lowest energy. The energy level *HOMO* − 1 is not listed here because it is degenerate with the *HOMO*; therefore, it has the same symmetry property as that of the *HOMO*. Thus, it would have the same transition property as that of *HOMO* as well. To verify the group theory results, we randomly choose 350 molecules and perform TD-DFT transition calculations in Gaussian 09. The result for the above molecule shows that excitations from the *HOMO* to the *LUMO* yield dark transitions, while excitations from *HOMO* − 2 to the *LUMO* yield bright transitions in only the z-direction, which agrees with our group theory calculation. The symmetry information in the QM-sym database provides the exact selection rules for the transition states of the molecules.

## Supplementary information

Supplementary Information.

## Data Availability

The newest version of database is available on Figshare and GitHub. Figshare 10.6084/m9.figshare.9638093. GitHub https://github.com/XI-Lab/QM-sym-database.
