# Supplementary material for: QM-sym, a symmetrized quantum chemistry database of 135 kilo molecules
Source: Sci Data. 2019 Oct 18;6:213. doi: 10.1038/s41597-019-0237-9 (PMC6802082; doi:10.1038/s41597-019-0237-9)
Supplement: Supplementary file 1 — Supplementary Information. [file 41597_2019_237_MOESM1_ESM.docx]

**Supplementary Information**

**QM-sym, A Symmetrized Quantum Chemistry Database of 135 Kilo Molecules**

Jiechun Liang, Yanheng Xu, Rulin Liu and Xi Zhu*

School of Science and Engineering, the Chinese University of Hong Kong Shenzhen,

Shenzhen, Guangdong, 518172, China

Email: [zhuxi@cuhk.edu.cn](mailto:zhuxi@cuhk.edu.cn)

Content:

Section S1. QM9 molecule figures and description


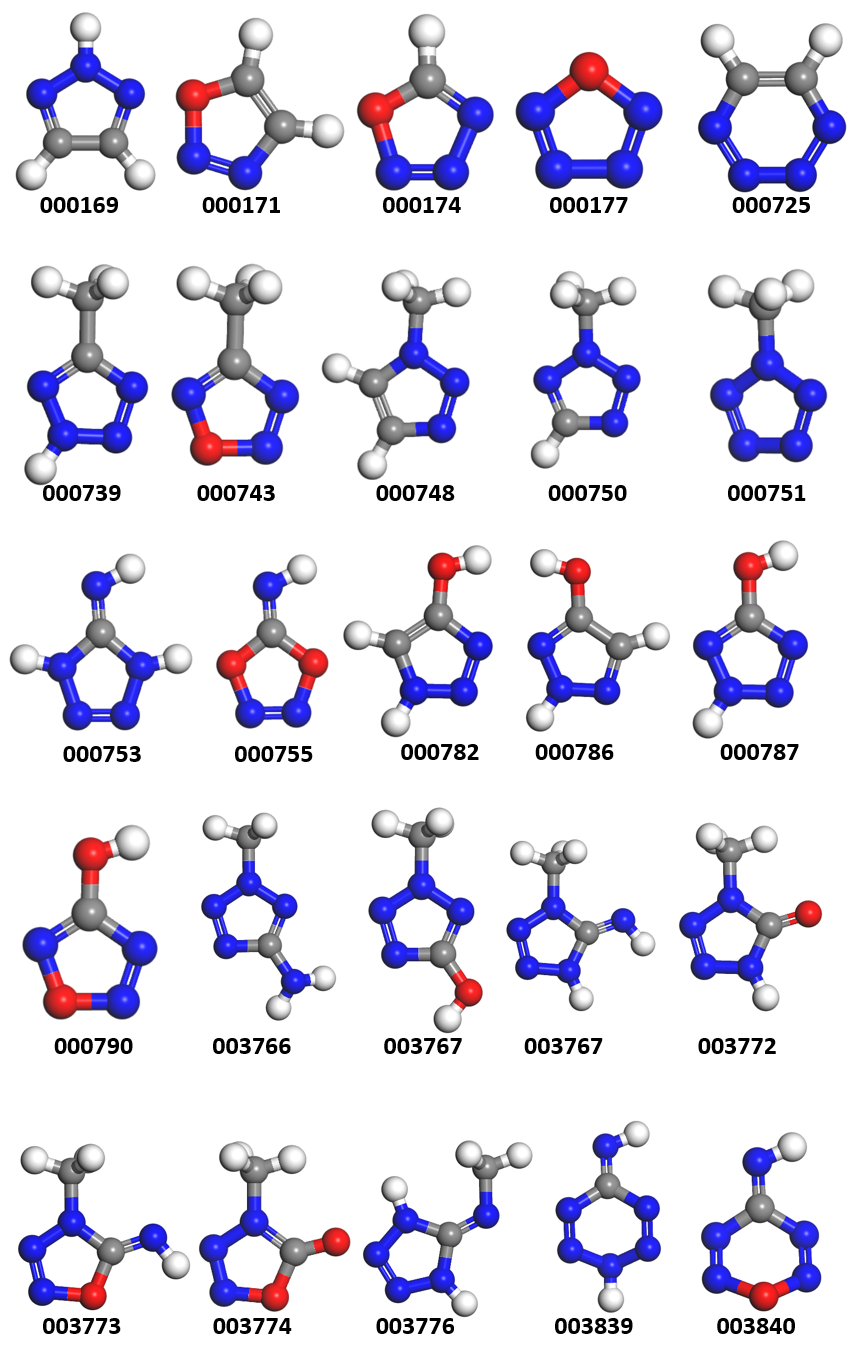
Section S1. QM9 molecule figures and description

Figure S1. Some selected suspiciously unstable molecules in QM9 database with their IDs. The blue, red, grey, and white balls denote nitrogen, oxygen, carbon, and hydrogen atoms. Because there are about 8k molecules and it is not suitable to put all figures in this supplementary information file, xyz files of these molecules are available on GitHub (https://github.com/XI-Lab/QM9-NN).
